# Direct Effects of HIV-1 Tat on Excitability and Survival of Primary Dorsal Root Ganglion Neurons: Possible Contribution to HIV-1-Associated Pain

**DOI:** 10.1371/journal.pone.0024412

**Published:** 2011-09-02

**Authors:** Xianxun Chi, Tohti Amet, Daniel Byrd, Kuei-Hua Chang, Kavita Shah, Ningjie Hu, Ayslinn Grantham, Sishun Hu, Jianhong Duan, Feng Tao, Grant Nicol, Qigui Yu

**Affiliations:** 1 Department of Pharmacology and Toxicology, Indiana University School of Medicine, Indianapolis, Indiana, United States of America; 2 Center for AIDS Research and Department of Microbiology and Immunology, Indiana University School of Medicine, Indianapolis, Indiana, United States of America; 3 Department of Chemistry and Purdue University Center for Cancer Research, Purdue University, West Lafayette, Indiana, United States of America; 4 Department of Anesthesiology and Critical Care Medicine, Johns Hopkins University School of Medicine, Baltimore, Maryland, United States of America; Emory University, United States of America

## Abstract

The vast majority of people living with human immunodeficiency virus type 1 (HIV-1) have pain syndrome, which has a significant impact on their quality of life. The underlying causes of HIV-1-associated pain are not likely attributable to direct viral infection of the nervous system due to the lack of evidence of neuronal infection by HIV-1. However, HIV-1 proteins are possibly involved as they have been implicated in neuronal damage and death. The current study assesses the direct effects of HIV-1 Tat, one of potent neurotoxic viral proteins released from HIV-1-infected cells, on the excitability and survival of rat primary dorsal root ganglion (DRG) neurons. We demonstrated that HIV-1 Tat triggered rapid and sustained enhancement of the excitability of small-diameter rat primary DRG neurons, which was accompanied by marked reductions in the rheobase and resting membrane potential (RMP), and an increase in the resistance at threshold (R_Th_). Such Tat-induced DRG hyperexcitability may be a consequence of the inhibition of cyclin-dependent kinase 5 (Cdk5) activity. Tat rapidly inhibited Cdk5 kinase activity and mRNA production, and roscovitine, a well-known Cdk5 inhibitor, induced a very similar pattern of DRG hyperexcitability. Indeed, pre-application of Tat prevented roscovitine from having additional effects on the RMP and action potentials (APs) of DRGs. However, Tat-mediated actions on the rheobase and R_Th_ were accelerated by roscovitine. These results suggest that Tat-mediated changes in DRG excitability are partly facilitated by Cdk5 inhibition. In addition, Cdk5 is most abundant in DRG neurons and participates in the regulation of pain signaling. We also demonstrated that HIV-1 Tat markedly induced apoptosis of primary DRG neurons after exposure for longer than 48 h. Together, this work indicates that HIV-1 proteins are capable of producing pain signaling through direct actions on excitability and survival of sensory neurons.

## Introduction

Globally, the HIV-1 (the causative agent of AIDS) pandemic has claimed over 25 million lives with 33.4 million people currently infected (2009 AIDS Epidemic Update by UNAIDS/WHO, www.unaids.org). The number of people living with HIV-1 worldwide continues to grow because of high rates of new infections and the beneficial impact of antiretroviral therapy (ART), also known as the highly active antiretroviral therapy (HAART). The vast majority (up to 90%) of these individuals living with HIV-1/AIDS have pain syndrome that has a significant impact on their psychological wellbeing and quality of life [1], [2], [3], [4], [5], [6], [7], [8], [9], [10], [11], [12]. Pain occurs at all stages of HIV-1 infection, although its severity and frequency are correlated with disease progression [1], [13]. Studies have reported that non-white patients (the majority of the HIV-1-infected people worldwide) are more likely than Caucasians to have uncontrolled pain when dying of HIV-1/AIDS [1], [14]. The most common pain syndromes in HIV-1/AIDS patients include painful peripheral neuropathies, headache, oral and pharyngeal pain, abdominal pain, chest pain, arthralgias and myalgias, painful dermatological conditions, and pain caused by HIV-1/AIDS-related malignancies such as Kaposi’s sarcoma [15], [16]. Given that patients with HIV-1/AIDS live longer with their illness in the era of HAART, which has successfully transformed HIV-1/AIDS from a death sentence to a chronic yet manageable disease for most individuals, management of symptoms including pain has been identified as one of the top priorities for HIV-1/AIDS clinical and translational research. However, pain is often under-assessed and undertreated in people with HIV-1/AIDS illness, and little progress has been made to address the issues of pain etiologies that are the key for pain management and improvement of the quality of life.

The pain in HIV-1/AIDS patients is usually divided into two categories: neuropathic and nociceptive [1], and has been suggested to be associated with (1) direct neurotoxic effects of viral components on neurons in both central and peripheral nervous systems, (2) immune dysregulation leading to inflammatory changes, opportunistic infections and tumors, and (3) drug-related adverse effects [1], [2], [16]. HIV-1 directly infects the perivascular macrophages [17], [18], resident microglia [19] and astrocytes [20], but not neurons in the central nervous system (CNS). While evidence of neuronal infection by HIV-1 is lacking, viral proteins are likely to be directly involved in neuronal damage and death. HIV-1 encodes a total of nine viral proteins including three structural proteins (Env, Pol, and Gag), two essential regulatory proteins (Tat and Rev), and four accessory proteins (Vif, Vpr, Vpu, and Nef). These viral proteins have been extensively studied for their role in HIV-1-associated CNS neuropathy. To date, five of these proteins including Env, Tat, Vpr, Nef, and Rev have been identified as potent neurotoxic viral proteins in that they directly induce neuronal cell death [17], [18], [19], [20], [21], [22], [23], [24], [25], [26]. These proteins are implicated in HIV-1-associated CNS pathologies such as mild to severe cognitive impairments (HIV-1-associated dementia) and encephalitis (NeuroAIDS) [17], [18], [19], [20], [21], [22], [23], [24], [25], [26]. However, the role of these proteins in the pathogenesis of HIV-1-associated pain has been largely unexplored. Recent studies have shown that Env and Vpr exert neurotoxic activities on peripheral sensory neurons (pain-sensing neurons). Injection of Env into the spinal intrathecal space of rats causes marked pain-like behavior [27], [28], [29], [30], [31], and Vpr enhances excitability of dorsal root ganglion (DRG) neurons [32]. These results suggest that HIV-1 neurotoxic proteins have direct effects on peripheral nerves, and may be causative factors in the generation of neuropathic pain in HIV-1-infected individuals. Notably, HIV-1 gp120 (surface unit of Env) appears to bind to the surface of rat DRG neurons, probably via the chemokine receptors CXCR4, CCR5 or/and others [33], and Tat appears to exert its neurotoxic activity via binding to the NMDA (*N*-methyl *D*-aspartate) receptor and the low-density lipoprotein receptor-related protein (LRP) [34], [35], [36], [37], whereas evidence of Vpr, Nef, or Rev binding to surface receptor(s) of neurons is lacking.

The current study assesses the direct effects of HIV-1 Tat protein on excitability and survival of rat primary DRG neurons. We demonstrated that Tat directly triggered rapid and sustained enhancement of the excitability of the small-diameter DRG neurons, probably by inhibiting kinase activity and mRNA production of Cdk5, a proline-directed serine/threonine kinase that has recently emerged as a key kinase in regulation of pain signaling in sensory neurons [38], [39], [40]. HIV-1 Tat also markedly reduced p35 (a Cdk5 activator) mRNA production and induced apoptosis of DRG neurons. These results indicate that Tat are capable of producing pain signaling through direct actions on excitability and survival of sensory neurons.

## Materials and Methods

### HIV-1 Tat and Gag proteins

Recombinant HIV-1_IIIB_ Tat and recombinant HIV-1_IIIB_ Gag (p24) were purchased from the Advanced Biotechnologies (Columbia, MD). These proteins were produced using the baculovirus expression system and purified by immunoaffinity chromatography. The proteins were reconstituted at appropriate concentrations in phosphate buffered saline (PBS), and then stored at −80°C until use. An aliquot of Tat protein was heat-inactivated by incubation at 85°C for 30 min as previously described [41]. The heat-inactivated Tat protein has been extensively used as a negative control for bioactive Tat [41], [42], [43] and was used as a control for the DRG excitability experiments in this study.

### Isolation and culture of adult rat sensory neurons

Primary DRG neurons were isolated from young adult male Sprague Dawley rats using procedures described in our previous report [44]. Briefly, young adult Sprague–Dawley rats (100 −150 g) (Charles River Laboratories, Wilmington, MA) were sacrificed by carbon dioxide narcosis according to the institutional protocol (the animal care and use protocol #3282 approved by the Institutional Animal Care and Use Committee of the Indiana University School of Medicine). DRG tissues were dissected from all spinal segments and were then collected in a culture dish filled with sterilized Puck's solution (without Ca^2+^ and Mg^2+^). The DRG tissues were transferred to a conical tube with Ham’s F-12 media (Mediatech, Manassas, VA) containing papain (20 U/ml) (Sigma Chemical, St. Louis, MO) and then incubated for 15 min at 37°C, followed by incubation in 1 mg/ml collagenase IA (Sigma Chemical, St. Louis, MO) and 2.5 mg/ml dispase (Sigma Chemical, St. Louis, MO) for 10 min. The DRG neurons were pelleted and were then resuspended in F-12 media supplemented with 30 ng/ml nerve growth factor (NGF, Invitrogen, Carlsbad, CA). The cells were mechanically dissociated with reduced bore pipettes until all obvious chunks of tissues were gone. The isolated cells were plated onto plastic coverslips or 6-well plates pre-coated with poly-d-lysine (100 µg/ml) and laminin (5 µg/ml), and were then cultured for 24–36 h at 37°C in a humidified atmosphere with 3% CO_2_. The cells were used for electrophysiological studies or others.

### Electrophysiology

Patch-clamp recordings were made at room temperature (23°C) using the whole-cell patch-clamp technique as described in our previous report [44]. Briefly, a coverslip with DRG neurons was placed in a recording chamber (Warner Instrument, Hamden, CT) where DRG neurons were bathed in normal Ringers solution of the following composition: 140 mM NaCl, 5 mM KCl, 2 mM CaCl_2_, 1 mM MgCl_2_, 10 mM HEPES, and 10 mM glucose (pH at 7.4). Recording pipettes were pulled from disposable borosilicate glass tubing and typically had resistances of 2–5 MΩ when filled with the following solution: 140 mM KCl, 5 mM MgCl_2_, 4 mM ATP, 0.3 mM GTP, 2.5 mM CaCl_2_, 5 mM EGTA (calculated free Ca^2+^ concentration of ∼100 nM), and 10 mM HEPES, at pH 7.3 with KOH. Whole cell voltages were recorded with an Axopatch 200B amplifier (Molecular Devices, Sunnyvale, CA). Data were acquired and analyzed using pCLAMP 8.2 or pCLAMP 9.0.2 (Molecular Devices, Sunnyvale, CA). The whole-cell recording configuration was established in normal Ringers solution. After establishing the whole-cell configuration, both the capacitance and resistance were compensated by ∼80%. To assess the excitability, the DRG neurons were held at their resting potentials (range between −42 and −62 mV), and a ramp of depolarizing current (1 s in duration) was applied to the neuron wherein the amplitude of the ramp was adjusted to produce three to four action potentials (APs) under control conditions. The stimulation amplitude then remained constant throughout the recording period for each individual neuron. The number of APs was recorded at 2, 4, 6, 10, and 15 min time points after exposure to the test agent using the same ramp amplitude. These recordings were sampled at 1–2 kHz using pClamp 8.0 (Axon Instruments, Weatherford, TX). To test whether the impact of Tat on DRG excitability is affected by roscovitine, Tat (20 µM) was focally applied to an individual DRG neuron to assess its actions over a 4 min time period, thereafter, the neuron was exposed to 10 µM roscovitine by external perfusion of the recording chamber. At the end of each recording, 100 nM capsaicin (a neurotoxin) was focally applied to the neuron for ∼2 seconds. If the neuron depolarized by at least 5 mV and generated APs, then it was judged to be capsaicin sensitive. Capsaicin has been used to distinguish capsaicin-sensitive sensory neurons that are believed to transmit nociceptive information [45], [46]. The electrophysiological results reported below were obtained from only capsaicin-sensitive small-diameter DRG neurons.

### Cdk5 kinase assay

Cdk5 kinase assays were conducted as described in our previous report [47], [48]. Briefly, DRG neurons treated with 20 µM of HIV-1 Tat or Gag from 15 min to 1 h were rinsed twice with cold PBS and lysed in 1% NP-40 lysis buffer (1% NP-40, 20 mM Tris, pH 8.0, 150 mM NaCl, 1 mM PMSF, 10 µg/ml leupeptin, and 10 µg/ml aprotinin) and cleared by centrifugation at 10,000 rpm for 10 min at 4°C. Cleared lysates were mixed with Cdk5 antibody and protein A Sepharose beads (Sigma Chemical, St. Louis, MO) and incubated at 4°C for 2 h. Immune complexes were washed twice with 1% NP-40 lysis buffer and twice with kinase buffer (50 mM Tris, pH 8.0, 20 mM MgCl_2_), and were then subjected to *in vitro* Cdk5 kinase assays using [γ-^32^P]ATP and 5 µg of Cdk5 substrate peptide (KHHKSPKHR) in a final volume of 30 µl buffered at pH 8.0 containing 50 mM Tris and 20 mM MgCl_2_ at 30°C. After 20 min of incubation, the reactions were terminated by spotting 25 µl of the reaction volume onto p81 phosphocellulose disks (Whatman, Clifton, NJ) and immersing in 100 ml of 10% acetic acid for 30 min, followed by three washings in 0.5% phosphoric acid (5 min each) and finally rinsing with acetone. The radioactivity was measured in a liquid scintillation counter. DRG neurons treated with PBS were included in each experiment as a control for obtaining baseline data.

### Semi-quantitative RT-PCR and real-time quantitative RT-PCR (qPCR)

Semi-quantitative RT-PCR and real-time quantitative RT-PCR (qPCR) studies were used to measure changes of Cdk5 and p35 mRNA abundance in DRG neurons in response to HIV-1 Tat, Gag or PBS treatment. Total RNA was isolated from DRG neurons after exposure to HIV-1 Tat, Gag or PBS using RNeasy Micro Kit (QIAGEN, Valencia, CA) according to the manufacturer's protocol. Prior to RNA elution from an RNeasy column, DNA removal was performed by on-column DNase digestion for 15 min at room temperature using RNase-Free Set (QIAGEN, Valencia, CA). Total RNA (1 µg per sample) were reverse transcribed into cDNA using the SuperScript First-Strand Synthesis System and oligo(dT)_12–18_ oligodeoxynucleotide primer (Invitrogen, Carlsbad, CA). Three plasmid constructs containing partial rat Cdk5, p35, or the house-keeping gene glyceraldehyde-3-phosphate dehydrogenase (GAPDH) gene were generated and used to establish standard curves in each real-time qPCR performance for quantitative analysis of cDNA copy number. RNA or plasmid DNA concentrations were determined using a ND-1000 UV–vis Spectrophotometer (NanoDrop Technologies, Wilmington, DE). The number of construct copies in the plasmid solution was calculated, based on plasmid vector size (pCR2.1-TOPO, Invitrogen, Carlsbad, CA) plus insert size of Cdk5, p35, or rat GAPDH. A plasmid-based calibration curve was generated with 10-fold serial dilutions of plasmid containing the Cdk5 or p35 gene fragment sequence; to control pipetting steps, three 10-fold serial dilutions were prepared, and concentrations were checked by real-time qPCR. For Cdk5 or p35, the quantification was linear over a range of 10 to 10^7^ starting plasmid copy numbers, and the detection limit was ten copies per reaction. The rat house-keeping gene GAPDH was used as an internal control for the normalization of the amount of cDNA in each sample.

Real-time qPCR was performed in a StepOnePlus Real-time PCR System (Applied Biosystems, Foster City, CA). Each sample and standard, including DRG neurons treated with HIV-1 Tat, Gag or PBS control, was set up in triplicate containing 5 µl of 2 × Sybr Green Master Mix, 15 pmol each primer, and 50–100 ng sample cDNA. PCR cycling conditions were 95 °C for 10 min, followed by 40 cycles of 95 °C for 30 s, 55 °C for 30 s, and 72 °C for 30 s, and then a final extension step at 72 °C for 10 min. The fluorescence intensity of SYBR green was measured automatically during the annealing steps. At the end of each run, a melting curve analysis was performed. Experiments were performed with undiluted and 10-fold diluted template cDNA in triplicate. The initial specimen cDNA was diluted in concentrations corresponding to 40,000 cell equivalents/10 µl with the number of cell equivalents calculated assuming that 6.6 ng of cDNA was equivalent to 1 × 10^3^ cells. Initially, RNA samples were adjusted to the same concentrations and the same amount of template was used for amplifying Cdk5 or rat GAPDH. The PCR primers used were described previously [49]. Semi-quantitative amplification was performed using the following primers: 5’-GGCACCTACGGAACTGTGTT-3′ (forward) and 5′-CACAATCTCAGGGTCCAGGT-3′ (reverse) for rat cdk5, 5′-TGACCTGTCTGTACCTCTCC-3′ (forward) and 5’-GCACAGAAAGTCATCAAAGCC-3′ (reverse) for rat p35, and 5′-GACATGCCGCCTGGAGAAAC-3′ (forward) and 5′-AGCCCAGGATGCCCTTTAGT-3′ (reverse) for rat GAPDH. For cdk5 real-time qPCR, the primers used were: forward 5′- AGCCTTTGGTATCCCAGTCC – 3′, and reverse 5′- TCCTCTTCAGCTGGTCATCC – 3′. Primers for rat p35 semi-quantitative RT-PCR were also used for p35 real-time qPCR.

### Flow cytometric analysis

Flow cytometric analysis was performed using a FACSCalibur (BD Biosciences, San Jose, CA) after staining by standard methods, and all data were analyzed using FlowJo software (Tree Star, San Carlos, CA). Apoptotic cell death and cellular apoptosis were determined by staining with annexin-V^FITC^ and propidium iodide (PI) from the annexin-V-FLUOS staining kit (Roche Applied Science, Indianapolis, IN) according to the manufacturer's protocol. Rat primary DRG neurons seeded in 6-well cell culture plates were incubated with 20 µM of HIV-1 Tat, Gag, or PBS for 30 min to 72 h. After incubation, the plates were chilled on ice and cells were gently scraped off the bottom of the plates with a rubber scraper. After washing twice with culture medium, cells were instantly subjected to staining with annexin-V^FITC^ and PI for analysis of apoptosis.

### Data analysis

All values obtained from electrophysiological studies represent the mean ± standard error of the mean (SEM). For the current-clamp study of excitability parameters, we measured resting membrane potential (RMP, mV), AP firing threshold (FT, mV), rheobase (pA) and the resistance at threshold (R_Th_, MΩ). The firing threshold was determined by differentiating the voltage trace (dV/dt) evoked by the ramp. The voltage at which the first AP was fired was taken as the point that exceeded the baseline value of dV/dt by >20-fold. The baseline dV/dt was determined by averaging the points between the onset and the following 100 ms of the ramp. The rheobase was measured as the amount of ramp current at the firing threshold. R_Th_ was calculated as the FT minus the RMP divided by the rheobase [R_Th_ = (RMP-FT)/rheobase]. Statistical differences between the control recordings and those obtained after treatment conditions were determined by using a one-way ANOVA, or a repeated measures ANOVA (RM ANOVA) wherever appropriate. When a significant difference was obtained with an ANOVA, a post-hoc analysis was performed using a Holm-Sidak all pairwise test or a Rank test. Values of *p*<0.05 were judged to be significant.

## Results

### HIV-1 Tat protein enhanced excitability of capsaicin-sensitive small-diameter DRG neurons

**Figure 1:**
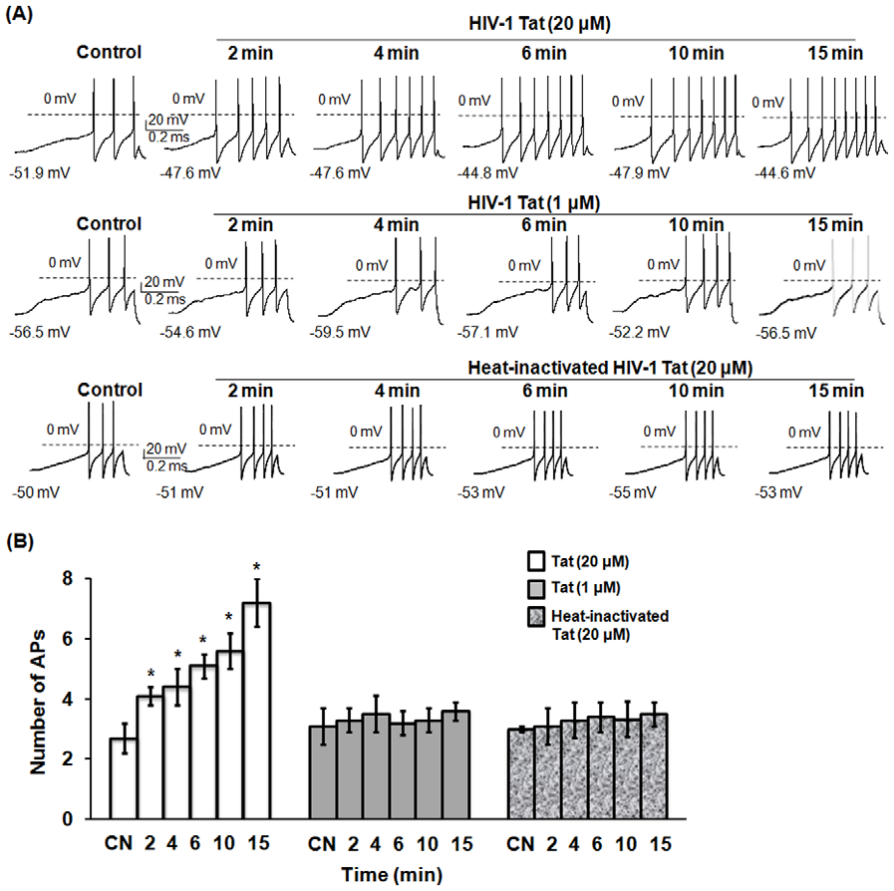
HIV-1 Tat enhanced excitability of small-diameter DRG neurons. Small-diameter rat primary DRG neurons were treated with HIV-1 Tat, Gag or heat-inactivated Tat, and then subjected to whole-cell patch-clamp recordings to determine action potentials (APs). (A) A representative experiment of AP recording from one of fifteen DRG neurons is shown. The experiment was repeated at least two times. DRG neurons were exposed to 1 µM or 20 µM of Tat, 20 µM of Gag or 20 µM of heat-inactivated Tat, and the APs were recorded at 2 to 15 min of post-exposure to viral proteins. Treatment with PBS was used as a control to obtain the baseline of APs. (B) Summary data from experiments performed on all fifteen DRG neurons are shown. Bars represent SEM on the mean. Comparisons were performed between PBS and all conditions according to time points of exposure using Anova or Repeated Measures Anova. Asterisk indicates statistically significant (*p<0.05) associations between PBS and Tat treatment according to time points of exposure. CN: control with PBS treatment, APs: action potentials.

HIV-1 infection is often associated with neurological complications including cognitive deficits such as HIV-1-associated dementia and milder forms such as cognitive/motor disorders. Uniquely, neuronal injury, cell loss and dysfunction during HIV-1 infection occur through soluble neurotoxins rather than productive virus infection. Of these soluble neurotoxins, HIV-1 Tat has been shown to be directly and indirectly neurotoxic in inducing neuronal dysfunction/toxicity, resulting in CNS pathology, such as the dementia and encephalitis associated with NeuroAIDS [50], [51]. To address whether HIV-1 Tat protein directly affects sensory neuron function leading to HIV-1-associated pain, we treated rat primary DRG neurons with Tat to study its effects on DRG excitability. Heat-inactivated Tat was included as a negative control. HIV-1 Gag protein does not elicit neurotoxicity and was included in this study as an additional control of a viral protein without neurotoxicity. The current-clamp configuration was used to examine the effects of HIV-1 Tat on the capacity of sensory neurons to fire action potentials (APs) when stimulated with a ramp of depolarizing current over time. As shown for a representative response (Fig. 1A), Tat, in a dose- and time-dependent manner, increased the number of APs recorded from small-diameter DRG neurons. While the amplitude of the ramp was adjusted to produce 3 - 4 APs under control conditions, exposure to 20 µM Tat for 2, 4, 6, 10 and 15 min produced 5, 6, 7, 7, and 8 APs, respectively (Fig. 1A), whereas exposure to 1 µM Tat appeared to have no effect on AP firing (yielding 3, 3, 3, 4, and 3 APs, respectively, Fig. 1A). In contrast, 1–20 µM of Gag treatment did not increase the number of APs when compared with controls (data not shown). Heat-inactivated Tat (20 µM) had no effect on the excitability of sensory neurons (Fig. 1A). The AP results of the current-clamp experiments obtained from a total of 15 small-diameter rat primary DRG neurons are summarized in Fig. 1B. Notably, Tat protein (20 µM) rapidly enhanced the excitability of DRG neurons. Within 2 min of exposure, Tat (20 µM) significantly increased the number of APs evoked by the current ramp from an average control value of 2.7±0.5 to 4.1±0.3 (n = 15, *p*<0.05). The number of APs increased with time of Tat exposure. At the 15 min time point, the number of APs increased to an average value of 7.3±0.8 when compared to the heat-inactivated Tat control (3.5±0.4) or ineffective concentration (1 µM) of Tat treatment (3.6±0.3). As shown in Table 1, the increase in AP firing triggered by Tat exposure at 20 µM was accompanied by a significant reduction in the rheobase at all time points examined from 2 to 15 min, and by a significant increase in R_Th_ at the time points of 10 and 15 min, and by a significant RMP depolarization at all time points examined from 2 to 15 min compared to the control values (*p*<0.01, RM ANOVA Holm-Sidak all pairwise test). However, Tat at 20 µM did not alter the FT at any time point examined. Although 1 µM Tat had no effect on AP, FT, R_Th_, and rheobase, this concentration of Tat produced a significant depolarization of RMP at the 10 min time point, suggesting that Tat at 1 µM may have a small biological effect on DRG neurons. Exposure to heat-inactivated Tat had no effect on parameters of DRG excitability including RMP, rheobase, FT, and R_Th_ at any time points examined from 2 to 15 min. To reduce the variance of the rheobase and R_Th_ measurements, these values were normalized to their respective control values (Table 1). After normalization, 20 µM Tat still showed a significant reduction in the rheobase at all time points examined from 2 to 15 min, and a significant increase in R_Th_ at the time points of 10 and 15 min. In contrast, neither 1 µM Tat nor heat-inactivated Tat had any significant effects on the values of the rheobase or R_Th_ over the 15 min treatment period (Table 1). These observations also demonstrate that the recordings of neuronal excitability are stable over this 15 min recording period. All of the small-diameter DRG neurons tested were sensitive to capsaicin as they depolarized by at least 5 mV and generated APs in response to capsaicin treatment (100 nM) at the end of each recording. Therefore, these results demonstrate that Tat causes hyperexcitability of sensory neurons, as it triggered rapid and sustained enhancement of AP firing that was accompanied by a significant reduction in the rheobase, an increase in R_Th_, and depolarization of the RMP.

**Table 1:** The effect of HIV-1 Tat on parameters of DRG excitability.

	Control	2 min	4 min	6 min	10 min	15 min
**Tat (20 μM)**						
RMP (mV)	−50±0.8	−48±0.8**	−48±0.8**	−47±0.3**	−46±0.4**	−45±0.4**
FT (mV)	−12.4±0.7	−13.1±0.8	−12.9±1.0	−12.9±1.0	−12.7±0.8	−11.8±0.9
Rheobase (pA)	306±39	252±32**	246±36**	219±23**	207±27**	213±30**
Normalized rheobase	1.0±0	0.84±0.04**	0.79±0.05**	0.75±0.05**	0.69±0.05**	0.70±0.05**
R_Th_ (MΩ)	146±55	162±62	181±81	177±68	194±84**	198±97**
Normalized R_Th_	1.0±0	0.84±0.04	0.79±0.05	0.75±0.05	0.69±0.05**	0.70±0.05**
**Tat (1 μM)**						
RMP (mV)	−54±1.2	−50±1.1	−53±1.6	−56±1.2	−49±0.8*	−51±1.2
FT (mV)	−16.3±1.9	−18.1±2.0	−19.7±2.1	−17.1±2.1	−15.7±1.8	−16.7±2.1
Rheobase (pA)	388±51	352±58	272±36	246±35	229±38	349±63
Normalized rheobase	1.0±0	0.91±0.08	0.74±0.11	0. 67±0.10	0.65±0.13	0.91±0.09
R_Th_ (MΩ)	104±10.7	105±16.6	139±27.7	192±49.3	192±49.7	114±18.3
Normalized R_Th_	1.0±0	0.84±0.04	0.79±0.05	0.75±0.05	0.69±0.05	0.70±0.05
**Inactivated Tat (μM)**						
RMP (mV)	−52±1.0	−51±1.1	−49±1.4	−50±1.1	−49±1.1	−51±1.4
FT (mV)	−11.9±1.5	−12.2±1.8	−13.4±1.7	−11.3±2.0	−10.6±2.5	−9.6±2.8
Rheobase (pA)	392±51	382±49	378±52	380±59	373±39	423±48
Normalized rheobase	1.0±0	0.99±0.06	0.99±0.08	0.99±0.10	1.01±0.09	1.11±0.06
R_Th_ (MΩ)	119±22	117±22	106±14	119±21	111±15	107±17
Normalized R_Th_	1.0±0	0.99±0.04	0.94±0.07	1.04±0.13	0.99±0.11	0.93±0.08

RMP: resting membrane potential; FT: firing threshold; R_Th_: resistance at threshold. Normalized rheobase: rheobase was normalized to the control. Normalized R_Th_: R_Th_ was normalized to the control. Data are expressed as mean ± S.E.M. N = 15.

*p<0.05;

**p<0.01. Groups were compared with controls by Anova or Repeated Measures Anova.

### HIV-1 Tat negatively regulated Cdk5 kinase activity and messenger RNA

**Figure 2:**
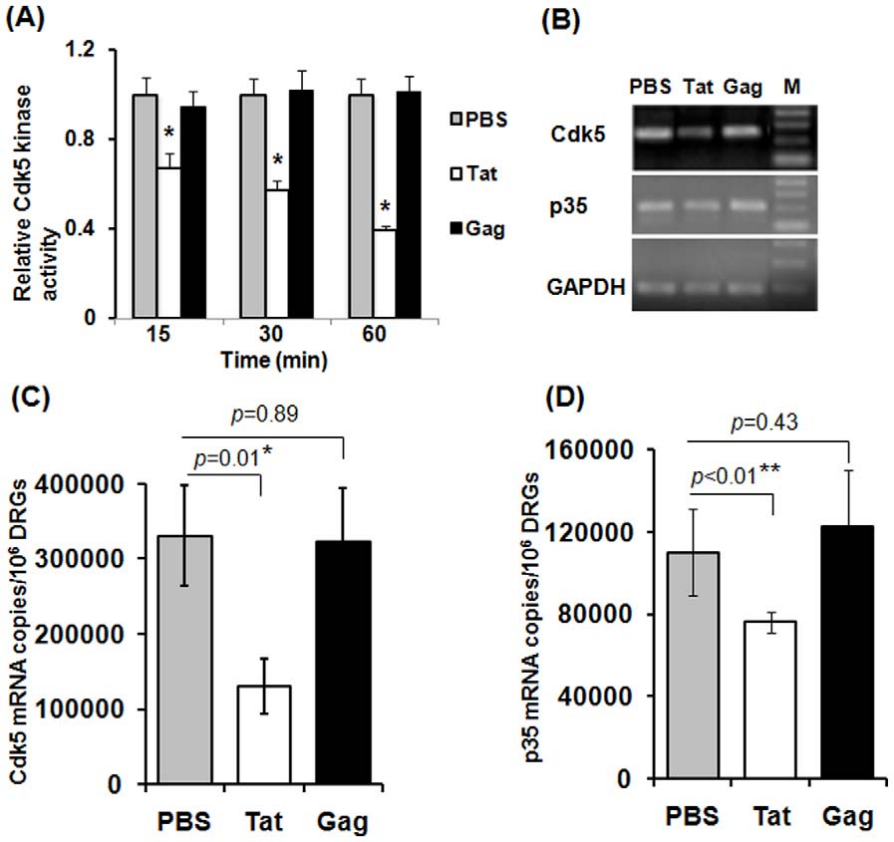
HIV-1 Tat inhibited endogenous Cdk5 kinase activity and mRNA production. (A) Cdk5 kinase activity in DRG neurons in response to exposure of HIV-1 Tat or Gag. Primary DRG neurons from rats were treated with 20 µM of HIV-1 Tat or Gag for various time intervals ranging from 15 min to 60 min, and were then subjected to Cdk5 kinase assays. Treatment with PBS was included to obtain the baseline of Cdk5 kinase activity. Open, solid gray, and solid black bars represent treatments of PBS, Tat, and Gag, respectively. Cdk5 kinase activity obtained from all conditions was normalized to PBS-treated controls. (B), (C) and (D) Direct effects of HIV-1 Tat and Gag on the regulation of Cdk5 mRNA and p35 mRNA levels in DRG neurons. DRG neurons were treated with 20 µM of HIV-1 Tat or Gag, or PBS for 4 h, and then subjected to analysis of Cdk5 or p35 mRNA production using (B) semi-quantitative RT-PCR and (C) and (D) real-time qPCR assays. Bars represent SD on the mean of three individual experiments. Asterisk indicates statistically significant (*p<0.05; **p<0.01) associations between PBS and Tat treatments.

Small-diameter DRG sensory neurons have central terminals in the dorsal horn of the spinal cord and peripheral terminals in skin, muscle, and other tissues. The hyperexcitability of these sensory neurons leads to abnormal burst activity that could underlie the enhanced pain sensitivity. HIV-1 Tat causes hyperexcitability of DRG neurons, suggesting that Tat exerts a direct effect on HIV-1-associated pain. Studies have shown that Cdk5, a proline-directed serine/theronine kinase, is most abundant in the nervous system including DRG neurons, and Cdk5 activity participates in the regulation of nociceptive signaling including pain [38], [39], [40]. We therefore studied whether HIV-1 Tat directly affected Cdk5 kinase activity and mRNA production. Primary DRG neurons from rats were treated with 20 µM of HIV-1 Tat or Gag protein for various time intervals ranging from 15 min to 60 min. Treatment with PBS was included to obtain the baseline of Cdk5 kinase activity and mRNA level. We found that HIV-1 Tat rapidly inhibited Cdk5 kinase activity as early as 15 min (Fig. 2A; 33.3% decrease). The inhibition effect was increased by 30 min (43% decreased), and further increased by 60 min (62% decreased). In contrast, HIV-1 Gag had no effect on Cdk5 activity (Fig. 2A).

We next examined the effect of HIV-1 Tat on the regulation of messenger RNA (mRNA) levels of Cdk5 and its activator p35. DRG neurons were treated with 20 µM HIV-1 Tat, Gag or PBS for 4 h, and then subjected to extraction of total RNA for semi-quantitative RT-PCR and real-time qPCR assays. As shown in Fig. 2B, a semi-quantitative RT-PCR assay revealed that Tat markedly reduced the levels of both Cdk5 and p35 mRNAs as compared with the PBS treatment. In contrast, treatment with HIV-1 Gag had no effect on the levels of Cdk5 or p35 mRNAs as compared with the PBS treatment. These results were further confirmed by real-time qPCR analysis. As shown in Fig. 2C and 2D, HIV-1 Tat significantly reduced Cdk5 cDNA copies (130,500±37,000 cDNA copies/10^6^ DRG neurons, n = 3) and p35 cDNA copies (76,098±5,234 cDNA copies/10^6^ DRG neurons, n = 3) as compared with PBS treatment (Cdk5: 330,800±66,400 cDNA copies/10^6^ DRG neurons, p35: 110,607±21,230 cDNA copies/10^6^ DRG neurons, n = 3). In contrast, Gag (Cdk5: 322,300±72,200 cDNA copies/10^6^ DRG neurons, p35: 122,839±27,256 cDNA copies/10^6^ DRG neurons, n = 3) only slightly affected Cdk5 and p35 cDNA copies, which was not statistically significant when compared with the PBS treatment. Thus, HIV-1 Tat, but not Gag, exerts an inhibitory effect on mRNA levels of both Cdk5 and p35.

### Cdk5 inhibitor enhanced the excitability of DRG neurons in a similar pattern to that triggered by HIV-1 Tat

To confirm that the suppression of Cdk5 kinase activity was involved in Tat-mediated DRG hyperexcitability, we used roscovitine (Sigma Chemical, St. Louis, MO), a potent inhibitor of Cdk5, to treat DRG neurons and then analyze DRG excitability. Beta-amyloid (β-amyloid) ^25–35^, the biologically active and highly toxic core fragment of full-length Ab (Ab1-42), was used as a Cdk5 activator to compare the effects of Cdk5 inhibition and activation on DRG excitability.

**Figure 3:**
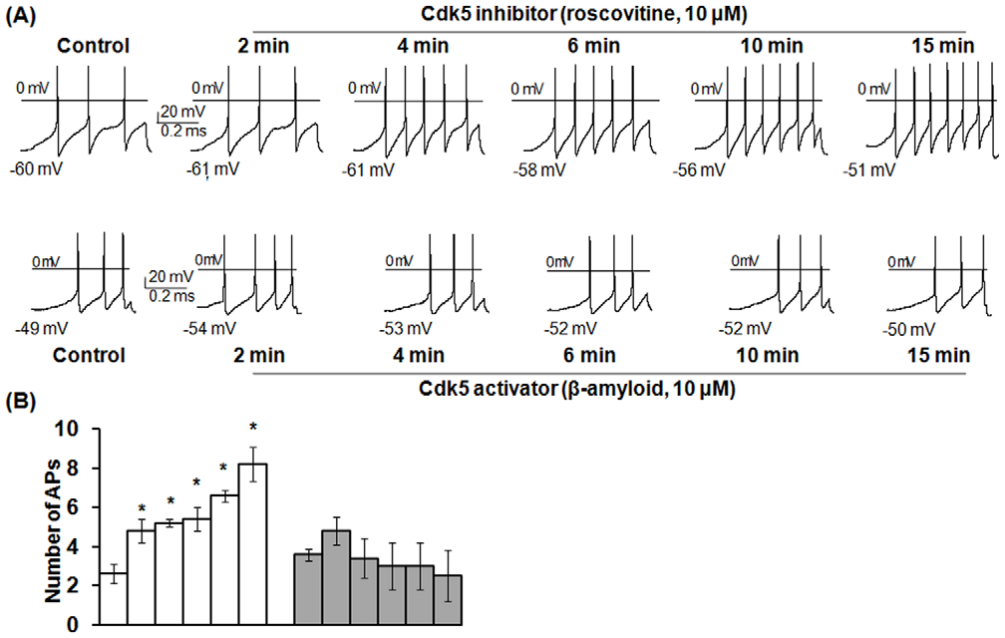
Inhibition of Cdk5 kinase activity enhanced the excitability of DRG neurons. Small-diameter rat primary DRG neurons were treated with Cdk5 inhibitor roscovitine (10 µM) or activator β-amyloid (10 µM), and then subjected to whole-cell patch-clamp recordings to determine action potentials (APs). (A) A representative experiment of AP recording from one of seven DRG neurons is shown. (B) Summary data from experiments performed on all seven DRG neurons are shown. Bars represent SEM on the mean. Comparisons were performed between PBS and roscovitine or β-amyloid according to time points of exposure using Anova or Repeated Measures Anova. Asterisk indicates statistically significant (*p*<0.05) associations between PBS and roscovitine or β-amyloid according to time points of exposure. CN: control with PBS treatment, APs: action potentials.

We first investigated the time- and concentration-dependent effects of the commonly used Cdk5 inhibitor roscovitine. We found that 10 µM or higher concentration of roscovitine rapidly enhanced the excitability of DRG neurons (Fig. 3A). Within 2 min of exposure, roscovitine (10 µM) significantly increased the number of APs evoked by the current ramp from an average control value of 2.6±0.3 to 4.8±0.2 (n = 5, *p*<0.05) (Fig. 3B). The number of APs increased with time of roscovitine exposure to 5.2±0.2, 5.4±0.6, 6.8±0.2, and 8.2±0.5 in response to exposure of roscovitine (10 µM) for 4 min, 6 min, 10 min and 15 min, respectively (Fig. 3B). As shown in Table 2, the increase in AP firing triggered by roscovitine exposure was accompanied by a significant reduction in the rheobase at the time points of 6 min, 10 min and 15 min and an increase in R_Th_ at the time points of 10 min and 15 min. To reduce the variance between the neurons, the rheobase and R_Th_ were normalized to their respective control values, these results are summarized in Table 2. Similar to Tat, roscovitine produced a significant depolarization of the RMP with no change in the FT (Tables 1 and 2). In contrast, treatment with 10 µM β-amyloid had no effect on the number of APs, RMP, RT, the rheobase, or R_Th_ over the 15 min recording period (Table 2). Thus, only the inhibitor of Cdk5, roscovitine, affected neuronal excitability. Notably, roscovitine causes DRG hyperexcitability in a very similar pattern to those triggered by Tat, suggesting Tat may use this signaling pathway to cause HIV-1 associated pain.

**Table 2:** The effect of Cdk5 inhibitor or activator on parameters of DRG excitability.

	Control	2 min	4 min	6 min	10 min	15 min
**Roscovitine**						
RMP (mV)	−52±1.3	−51±1.8	-47±2.4**	−46±2.1**	−44±2.0**	−43±1.4**
FT (mV)	−13.8±0.9	−12.9±1.1	−13.4±0.8	−12.9±2.9	−13.3±1.6	−11.9±1.3
Rheobase (pA)	209±26	202±28	181±25	131±25**	92±18**	77±14**
Normalized rheobase	1.0±0	0.95±0.03	0.86±0.04*	0.62±0.07**	0.43±0.05**	0.36±0.04**
R_Th_ (MΩ)	199±22	209±26	202±24	292±43	383±50**	458±58**
Normalized R_Th_	1.0±0	1.04±0.03	1.02±0.05	1.49±0.21	1.93±0.20**	2.33±0.25**
**ß-Amyloid**						
RMP (mV)	−51±0.5	−50±2.4	−50±1.5	−52±2.8	−49±1.4	−49±1.1
FT (mV)	−12.3±1.9	−13.0±1.4	−13.1±1.8	−11.3±1.7	−11.8±1.8	−13.4±2.0
Rheobase (pA)	276±38	268±47	286±33	290±30	299±36	307±43
Normalized rheobase	1.0±0	0.95±0.04	1.08±0.13	1.10±0.14	1.12±0.12	1.15±0.13
R_Th_ (MΩ)	153±25	153±23	135±14	148±21	130±14	126±17
Normalized R_Th_	1.0±0	1.02±0.08	0.94±0.11	1.01±0.13	0.89±0.09	0.86±0.11

RMP: resting membrane potential; FT: firing threshold; R_Th_: resistance at threshold. Normalized rheobase: rheobase was normalized to the control. Normalized R_Th_: R_Th_ was normalized to the control. Data are expressed as mean ± S.E.M. N = 7.

*p<0.05;

**p<0.01. Groups were compared with controls by Anova or Repeated Measures Anova.

### Tat-mediated increase of AP and depolarization of RMP were not affected by roscovitine

**Figure 4:**
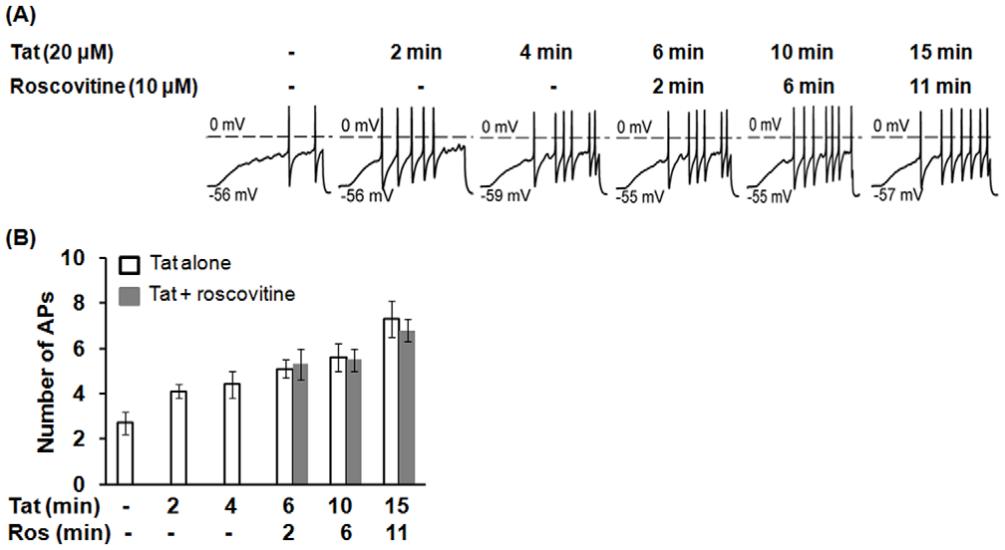
Tat-mediated increase of AP and depolarization of RMP were not affected by roscovitine. Tat at 20 μM was focally applied to an individual DRG neuron to assess its actions over a 4 min time period, thereafter, the neuron was exposed to 10 μM roscovitine by external perfusion of the recording chamber. The whole-cell patch-clamp recordings were used to determine APs prior to Tat exposure (control), exposure to Tat or roscovitine or both. (A) A representative experiment of AP recording from one of five DRG neurons is shown. (B) Summary data from time points of Tat alone (n = 15) and Tat plus roscovitine co-exposure performed on all five DRG neurons are shown. Bars represent SEM on the mean. Ros: roscovitine, APs: action potentials.

**Figure 5:**
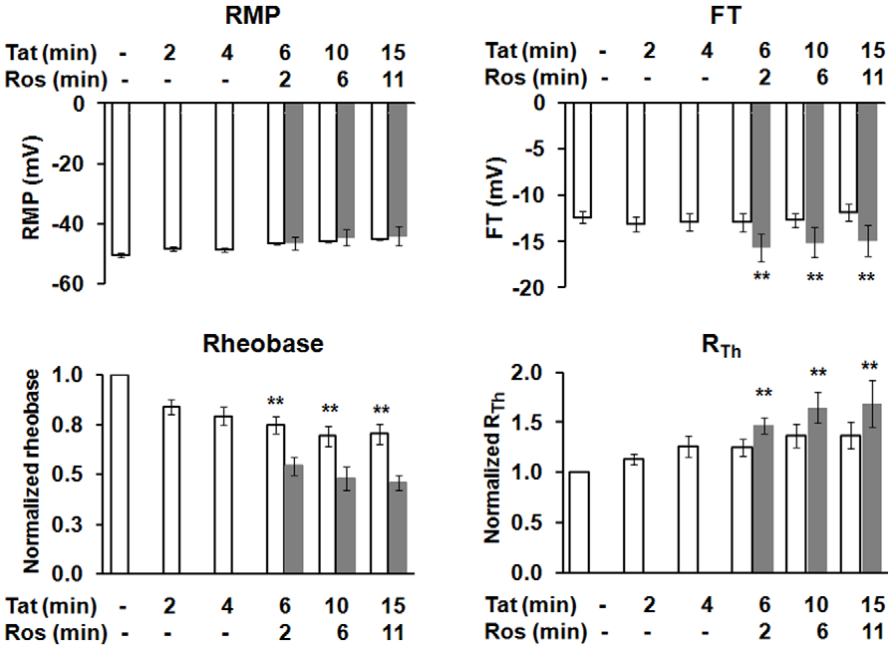
Comparison of parameters of DRG excitability between treatments of Tat alone verse Tat then roscovitine. Parameters of DRG excitability including RMP, FT, Rheobase and R_Th_ were compared between groups of Tat treatment alone versus Tat pretreatment followed by roscovitine exposure. The rheobase and R_Th_ were normalized to their respective control values. Bars represent SEM on the mean. Asterisk indicates statistically significant (*p<0.05; **p<0.01) associations between the two groups at the same time point using Student T-test. Open bars: Tat alone, solid gray bars: Tat plus roscovitine. Ros: roscovitine, APs: action potentials, RMP: resting membrane potential; FT: firing threshold; and R_Th_: resistance at threshold.

Tat caused DRG hyperexcitability in a very similar pattern to that triggered by Cdk5 inhibitor roscovitine, suggesting that Tat and roscovitine may affect bioactivity of DRG neurons via the same or overlapping intracellular pathways. To test this possibility, 20 μM Tat was focally applied to individual neurons for an exposure time of 4 min. At this point the neuron was additionally exposed to 10 μM roscovitine by bath perfusion. A representative experiment (Fig. 4A) and summary data from 5 small-diameter rat primary DRG neurons (Fig. 4B) demonstrated that Tat-mediated increase of AP was not affected by subsequent addition of roscovitine. Similarly, Tat-mediated depolarization of RMP was not affected by roscovitine (Fig. 5 and Table 3), as Tat plus roscovitine treatment depolarized RMP to -46.4 ± 2.2 (n = 5), −44.4 ± 2.7 (n = 5), and −44.0 ± 3.3 (n = 5) at the time points of 6 min, 10 min, and 15 min, respectively, which were not different for the depolarization of RMP produced by Tat alone at these time points (6 min: −46.6 ± 0.3, n = 15, *p* = 0.72 *t*-test; 10 min: −45.8 ± 0.4, n = 15, *p* = 0.05 *t*-test, and 15 min: −45.1 ± 0.4, n = 15, *p* = 0.20 *t*-test) (Fig. 5 and Table 3). However, roscovitine significantly accelerated the effects of Tat on the rheobase and R_Th_ (Fig. 5 and Table 3). Tat plus roscovitine significantly reduced the normalized value of the rheobase further to 0.54 ± 0.04 (n = 5), 0.48 ± 0.06 (n = 5), and 0.46 ± 0.04 (n = 5) at the time points of 6 min, 10 min, and 15 min, respectively, when compared with that produced by Tat alone at these time points (6 min: 0.75 ± 0.05, n = 15, *p*<0.01 *t*-test; 10 min: 0.69 ± 0.05, n = 15, *p*<0.01 *t*-test, and 15 min: 0.70 ± 0.05, n = 15, *p*<0.01 *t*-test) (Fig. 5 and Table 3). Meanwhile, Tat plus roscovitine significantly increased the normalized value of R_Th_ further to 1.47 ± 0.08 (n = 5), 1.65 ± 0.16 (n = 5), 1.68 ± 0.24 (n = 5) at the time points of 6 min, 10 min, and 15 min, respectively, when compared with that produced by Tat alone at these time points (6 min: 1.25 ± 0.09, n = 15, *p*<0.01 *t*-test; 10 min: 1.37 ± 0.11, n = 15, *p*<0.01 *t*-test, and 15 min: 1.37 ± 0.13, n = 15, *p*<0.01 *t*-test) (Fig. 5 and Table 3). Thus, these results demonstrate that Tat-mediated increase of AP and depolarization of RMP were not affected by roscovitine, but the effects of Tat on the rheobase or R_Th_ were accelerated by roscovitine, suggesting that Tat-mediated changes in DRG excitability are partly facilitated by Cdk5 inhibition.

**Table 3:** Comparison of parameters of DRG excitability between treatments of Tat alone verse Tat then roscovitine.

	Control	2 min	4 min	6 min	10 min	15 min
**Tat (20** µ**M)**		**+**	**+**	**+**	**+**	**+**
**Roscovitine (10** µ**M)**				**+ (2** **min)**	**+ (6** **min)**	**+ (11** **min)**
RMP (mV)	−53±1.3	−50±1.6	−50±2.8	−46±2.2**	−44±2.7**	−44±3.3**
FT (mV)	−14.6±1.8	−15.0±2.2	−14.3±2.3	−15.6±2.3	−15.1±1.6	−14.9±2.1
Rheobase (pA)	348±66	281±52	219±46**	179±25**	154±20**	152±20**
Normalized rheobase	1.0±0	0.82±0.04**	0.63±0.06**	0.54±0.04**	0.48±0.06**	0.46±0.04**
R_Th_ (MΩ)	125±22	148±35	184±35	186±39	208±47	222±70**
Normalized R_Th_	1.0±0	1.13±0.09	1.45±0.05	1.47±08	1.65±0.16**	1.68±0.24**

RMP: resting membrane potential; FT: firing threshold; R_Th_: resistance at threshold. Normalized rheobase: rheobase was normalized to the control. Normalized R_Th_: R_Th_ was normalized to the control. Data are expressed as mean ± S.E.M. N = 5.

*p<0.05;

**p<0.01. Groups were compared with controls by Anova or Repeated Measures Anova.

### HIV-1 Tat induced apoptosis of DRG neurons

**Figure 6:**
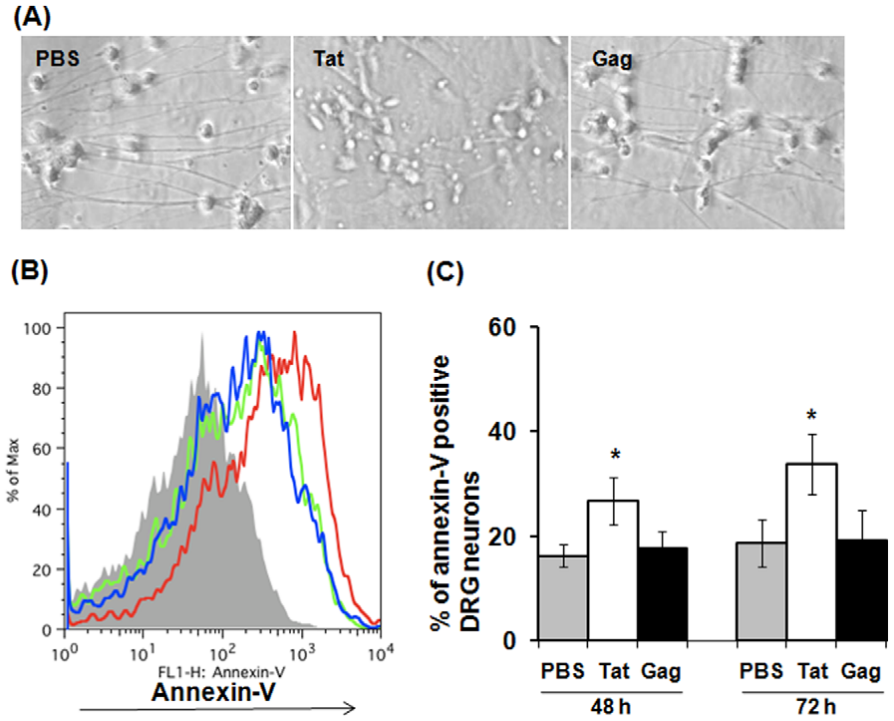
DRG neurons underwent apoptosis in response to chronic exposure of HIV-1 Tat. Rat primary DRG neurons were exposed to 20 µM of HIV-1 Tat or Gag for 30 min to 72 h, and were then subjected to apoptosis analysis by Annexin-V staining and flow cytometric analysis. (A) DRG morphological changes in response to exposure of HIV-1 proteins for 48 h. (B) A representative annexin-v staining of DRG apoptosis in response to exposure of HIV-1 Tat or Gag for 48 h. Solid gray histogram represents a staining control without adding annexin-V^FITC^, whereas histograms with blue, green, and red lines represent annexin-V staining on DRG neurons treated with PBS, Gag, and Tat, respectively. (C) Summary data from three independent experiments of annexin-V staining on DRG neurons in response to exposure of HIV-1 Tat and Gag for 48 h or 72 h are shown. Bars represent SD on the mean of three individual experiments. Asterisk indicates statistically significant (*p*<0.05) associations between PBS and Tat or Gag according to time points of exposure.

HIV-1 Tat has been implicated in inducing apoptosis of neurons in the CNS. To determine whether HIV-1 Tat also induces apoptosis in rat DRG neurons, we exposed isolated neurons to 20 µM Tat for 30 min to 72 h and then assessed the extent of apoptosis by Annexin-V staining and flow cytometric analysis. Treatment with 20 µM HIV-1 Gag was performed as a control. Treatment with HIV-1 Tat for longer than 48 h induced DRG morphology changes and apoptosis (Fig 6A, 6B and 6C) whereas exposure for less than 48 h only enhanced apoptosis slightly, but did not show a significant difference when compared to PBS treatment (data not shown). In contrast, HIV-1 Gag did not affect rat DRG survival as annexin staining results did not change when compared with that of PBS-treated DRG neurons (Fig 6A, 6B and 6C). These findings also correlated with viable cell counts, as determined by trypan blue exclusion (data not shown). Therefore, chronic exposure of HIV-1 Tat triggers apoptosis of DRG neurons *in vitro*.

## Discussion

Small-diameter DRG neurons have central terminals in the spinal cord dorsal horn and peripheral terminals in skin, muscle, and other peripheral tissues. These DRG neurons transmit and relay pain-related signals and temperature sensation from peripheral tissues to the spinal cord and brain. Neurotoxins and inflammatory molecules can cause hyperexcitability of DRG neurons leading to spontaneous or persistent firing. These agents can also induce apoptosis of DRG neurons, resulting in permanent neuron damage, death, and even nerve lesions. It is well established that peripheral sensitization of primary DRG neurons is the key event in the onset of chronic pain conditions [52]. Peripheral sensitization is defined as the enhancement of a baseline response, such as AP firing, after exposure to a defined mediator [52]. For example, exposure to the pro-inflammatory prostaglandin E_2_ (PGE_2_), increases the number of APs evoked by an excitatory stimulus by about three-fold compared to the control, thus the neuronal output is intensified by PGE_2_ [53], [54]. This increased firing is believed to be critical in the enhanced perception of pain sensation for both inflammatory and neuropathic conditions [53], [54]. Several HIV-1 proteins, in particular Tat, exhibit neurotoxic properties and therefore may directly impact excitability and survival of small-diameter DRG neurons, leading to HIV-1-associated pain. In this study, we show that HIV-1 Tat, but not Gag or heat-inactivated Tat, triggers rapid and sustained enhancement of the excitability of small-diameter rat primary DRG neurons. The Tat-mediated increase in DRG excitability was accompanied by marked reductions in the rheobase and RMP, and an increase in R_Th_. This Tat-mediated rapid onset of hyperexcitability of DRG neurons may play a key role in the initiation of HIV-1-associated pain, particularly during the early stages of the viral infection because (1) HIV-1-associated pain occurs even before severe neuronal injury and death. Pain has been described at all stages of HIV-1 infection, although its severity and frequency correlate with disease progression [1], [13]. Severe neuronal injury and death generally occurs in later or advanced stages of HIV-1 infection, and thus is unlikely to be responsible for pain during the early stages of viral infection. (2) Patients in the early stages usually have not yet received ART, and therefore have high levels of HIV-1 proteins in their circulations due to the burst of rapid viral replication and of viral protein secretion. In the early stages of HIV-1 infection, viral loads in both plasma and cerebrospinal fluid (CSF) are very high, commonly approaching several million viruses per mL [55], [56].

The concentration of Tat required to induce hyperexcitability of DRG neurons in this study is much higher than that in the sera of HIV-1 patients. Soluble Tat levels in the sera of HIV-1-infected patients have been measured up to 40 ng/ml (∼ 30 nM) [57], [58]. However, Tat concentrations surrounding HIV-infected cells are much higher than sera concentrations because the interactions of Tat with endogenous glycosaminoglycans and heparin sulfates lower measurable Tat concentration *in vivo* [58], [59], [60]. In addition, genetically engineered recombinant Tat is considerably less potent than Tat released from infected cells, suggesting that comparatively higher doses of the recombinant Tat are required to adequately mimic the effects of Tat released from HIV-1-infected cells [35]. Moreover, human sensory neurons may be more sensitive to Tat than that of rats, making it possible that a lower concentration of Tat may induce excitability of human sensory neurons. Finally, in the context of HIV-1 infection, the effects of Tat may occur over long-term chronic low-dose exposure. Therefore, our data must be validated by *in-vivo* studies such as injection of Tat into the spinal intrathecal space of rats to study pain-like behavior.

Tat also triggered apoptosis of DRG neurons in a pattern of chronic exposure, which is in accordance with the chronic effects of HIV-1 infection on nerve degeneration. Such chronic effects of Tat on survival of DRG neurons may contribute to neuronal injury and death in late or advanced stages of HIV-1 infection, resulting in more severe and frequent of pain with disease progression. Thus, HIV-1 Tat may be involved in pain at all stages of HIV-1 infection, i.e. the rapid onset of DRG hyperexcitability triggered by Tat may be mainly responsible to pain at very early stages of HIV-1 infection, whereas Tat-mediated chronic apoptosis of sensory neurons causes and accelerates pain in the advanced stages of the disease.

Previous reports have demonstrated that Cdk5, a proline-directed serine/threonine kinase, is most abundant in DRG and other neurons, and its kinase activity and protein expression can be altered in response to peripheral inflammation, resulting in enhancement of pain signaling [38], [39], [40]. We thus speculated that Tat might mediate DRG hyperexcitability by regulating Cdk5 kinase activity. We found that HIV-1 Tat caused an immediate inhibition of Cdk5 kinase activity and down-regulation of Cdk5 mRNA production, whereas HIV-1 Gag had no effects. In addition, we found that Tat also inhibited p35 (an activator of Cdk5) mRNA production in DRG neurons, which is in agreement with previous results [61], [62]. Thus, Tat employs dual mechanisms to inhibit Cdk5 activity, i.e., directly inhibits Cdk5 kinase activity and its mRNA production and indirectly inhibits Cdk5 activity by suppressing Cdk5 activator p35 mRNA production. However, Tat-mediated DRG hyperexcitability may be related to direct inhibition of Cdk5 kinase activity rather than suppression of Cdk5 and p35 mRNA or protein production because of the rapid onset of DRG hyperexcitability in response to Tat treatment. Rapid Cdk5 inhibition upon HIV-1 Tat treatment was coupled with rapid DRG hyperexcitability, which suggested that there might be a relationship between the two events. To investigate this relationship, we used roscovitine, a potent inhibitor of Cdk5, to treat DRG neurons. We also used β-amyloid, a biological activator of Cdk5, as a control to compare the effects of a Cdk5 inhibitor and activator on DRG excitability. We found that β-amyloid did not affect DRG excitability. In contrast, roscovitine induced a very similar pattern of DRG hyperexcitability to that triggered by Tat: an immediate increase of AP numbers, which was accompanied by marked reductions in the rheobase and RMP, and an increase in R_Th_. Furthermore, pre-application of Tat prevented roscovitine from having additional effects on the RMP and APs. These results suggest that Tat-mediated hyperexcitability of DRGs may be facilitated by Cdk5 inhibition. However, the effects of Tat on the rheobase and R_Th_ were accelerated by roscovitine, indicating that the intracellular pathways affected by Tat and roscovitine may not fully overlap. It is also possible that roscovitine accelerated the effects of Tat on the rheobase and R_Th_ by its side effects, as this chemical compound is also a potent inhibitor of CDK2 and other kinases [63]. We are currently studying the molecular mechanisms of Tat-mediated suppression of Cdk5 kinase activity and gene expression.

Cdk5 has recently emerged as an essential kinase in sensory pathways and its activity is tightly regulated. Previous studies have shown that p35-overexpressing transgenic mice, with elevated levels of Cdk5 activity are hypersensitive to painful thermal stimuli [38], [39], [40]. In contrast, our data using HIV-1 Tat protein revealed that aberrant inhibition of Cdk5 activity triggers pain signaling. Furthermore, elevated levels of Cdk5 activity have been associated with an increase of apoptosis in cultured neuronal cells [64]. We show that Cdk5 inhibition also promotes apoptosis of DRG neurons. These distinct results reveal that both aberrant activation or inhibition of Cdk5 signaling can promote pain signaling and neuronal cell death, indicating Cdk5 as a master switch controlling neuronal survival and death [65], [66], [67], [68]. Thus, basal Cdk5 activity is important for maintaining normal functions and survival of DRG neurons.

Tat and gp120 can be actively released by HIV-1-infected cells into the circulating blood stream, the extracellular space, and cerebrospinal fluid (CSF) [50], [59], [69], [70]. These two viral proteins have been demonstrated to directly induce apoptosis of neurons in the CNS, possibly through different mechanisms. Soluble HIV-1 gp120 can bind CXCR4, CCR5 and/or others on neurons in the absence of CD4 [71], [72], [73], [74]. The lethal signal transduction may involve a number of kinases, transcription factors, and typical ingredients of the mitochondrial apoptotic cascade [72]. Some of these mechanisms may also be used by gp120 to induce apoptosis of sensory neurons including DRGs [31], [75], [76]. HIV-1 Tat induces apoptosis of neurons [77], [78], [79], [80], [81], [82], [83], [84], however, by binding to the low-density lipoprotein receptor-related protein (LRP) with subsequent activation of the Ca^2+^-permeable NMDA receptor (NMDAR) [37], [85]. Activation of NMDAR allows the flow of Ca^2+^ and other ions into the cells, resulting in activation of neuronal nitric oxide synthase (nNOS) causing cell apoptosis [37], [85], [86]. Recent studies also demonstrate that Tat directly binds to NMDAR leading to excitotoxicity [34], [36], [87], [88], [89], [90]. The HIV-1 Tat protein is an 86 – 101 amino acid (aa) protein encoded by two exons. The most functional properties of Tat are attributed to Tat 1-72 encoded by exon 1. Tat 1-72 has an essential cysteine (Cys) rich region that mediates the interaction of NMDAR [35]. This Cys-rich region contains a Cys30-Cys31 motif that is critical for exciting the NMDAR as the mutation of Cys31 significantly attenuate Tat neurotoxicity [35]. Interestingly, Cdk5 and the NMDAR are known to physically and functionally associate with each other, as (1) anti-Cdk5 antibodies coprecipitate NMDAR from brain extracts [91], indicating that the two proteins bind to each other, and (2) Cdk5 kinase has been shown to direct or indirectly modulate NMDAR function by phosphorylating the long tail of an NMDAR subunit [92], [93]. Here, we demonstrate for the first time that HIV-1 Tat induces hyperexcitability and apoptosis of DRG neurons, probably by inhibiting Cdk5 kinase activity and protein expression. Thus, it will be exciting to determine which region or motif of Tat mediates DRG neurotoxicity and whether Tat exerts its neurotoxicity through a chain reaction of NMDAR-Cdk5 interaction.

Taken together, our study demonstrates that HIV-1 Tat causes hyperexcitability and apoptosis of DRG neurons, probably by inhibiting Cdk5 kinase activity and protein expression. Tat-mediated hyperexcitability of DRG neurons may play a key role in the initiation of HIV-1-associated pain in patients at the early stages of the viral infection. HIV-1 Tat and other proteins induce apoptosis of DRG neurons, which may be involved in HIV-1-associated pain at all stages of viral infection. This study has potentially important implications for developing therapeutic strategies to prevent or treat HIV-1-assciated pain. For example, early initiation of HAART and/or neutralization of HIV-1 protein neurotoxicity may significantly prevent or delay HIV-1-associated pain. A recent study using a simian immunodeficiency virus (SIV)-macaque model of HAART demonstrates that early initiation of HAART results in a dramatic reduction of viral RNA levels in plasma, CSF and brain [94], suggesting that early initiation of HAART can reduce or delay neurological complications including pain in HIV-1-infected patients.
